# Radical-initiated alkene hydroauration as a route to gold(iii) alkyls: an experimental and computational study†

**DOI:** 10.1039/c7ra13481a

**Published:** 2018-01-12

**Authors:** Anna Pintus, Manfred Bochmann

**Affiliations:** School of Chemistry, University of East Anglia Norwich Research Park Norwich NR4 7TJ UK m.bochmann@uea.ac.uk +44(0)-1603-592044

## Abstract

The hydroauration of functionalised 1-alkenes by the gold(iii) hydride (C^N^OMe^^C)AuH is initiated by organic radicals and proceeds *via* (C^N^C)Au(ii) radical intermediates following a bimolecular outer-sphere mechanism. The outcome of these reactions is determined by the stability of the gold-substituted radicals, and chemoselectivity correlates with the degree of spin delocalisation in the alkylgold radical intermediates. The reaction is sensitive to steric as well as electronic factors; disubstituted alkenes and alkenes that form unstable radicals give product mixtures or are unreactive. As DFT calculations show, the reactions agree well with the calculated reaction enthalpies and the standard free energy change for the reaction of the gold(ii) radical with the respective alkene.

## Abstract 2

Gold(iii) hydrides undergo radical-induced insertion reactions with alkenes *via* gold(ii) intermediates. The success of the reaction depends on the degree of spin delocalisation.

## Graphical abstract

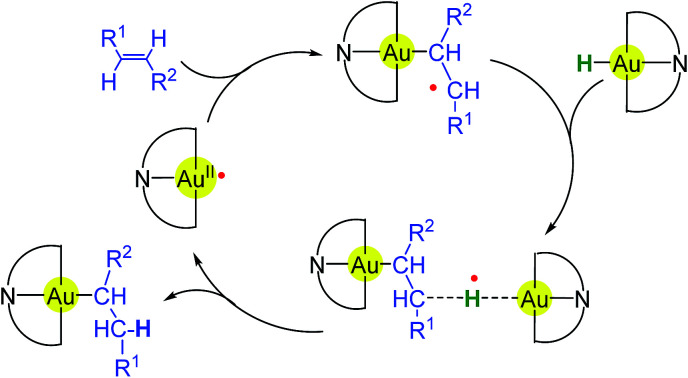

## Introduction

**Scheme 1:**
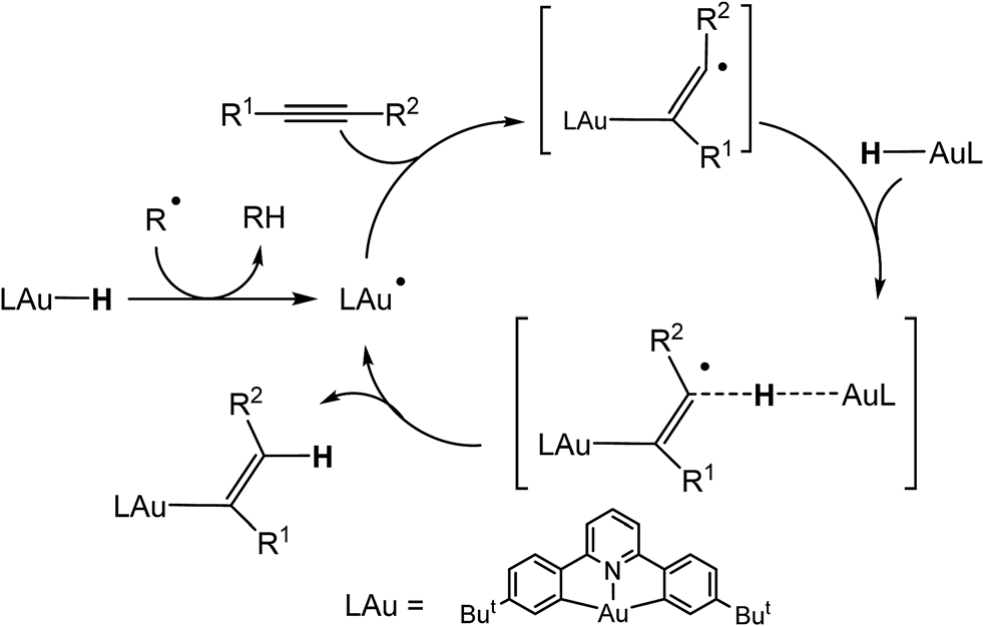
Mechanism of radical-mediated alkyne hydroauration with (C^N^C)AuH pincer complexes.^8^

We recently reported the synthesis of the first example of a gold(iii) hydride complex (C^N^C)AuH, based on the stabilisation provided by a C^N^C pincer ligand framework [(C^N^C) = 2,6-(C_6_H_3_Bu^*t*^)_2_pyridine].^1^ Cyclometallated C^N^C pincer complexes of gold(iii)^2,3^ have proved particularly useful for the stabilisation of otherwise non-isolable species, including gold(iii) alkene,^4^ alkyne,^5^ CO^6^ and peroxide complexes.^7^ (C^N^C)AuH proved to be thermally stable and did not react with air, moisture or even acetic acid and was also unreactive to alkenes and alkynes. On the other hand, it did react with allenes to give gold vinyl complexes in high yield.^1^ This lack of reactivity is not entirely unsurprising: gold(iii) adheres strictly to a square-planar coordination geometry and in (C^N^C)AuH all four coordination sites are occupied, so that these pincer compounds lack the ability to bind unsaturated substrates. However, we discovered that alternative reaction pathways become accessible in the presence of traces of organic radicals capable of abstracting the hydrogen ligand and thus generating (C^N^C)Au(ii)˙ radical species. These Au(ii) radicals can readily bind alkynes and lead to alkyne hydroauration in a bimolecular outer-sphere process (Scheme 1). Increasing the concentration of radicals greatly increases the rate of insertion reactions into Au–H bonds. This pathway allows the hydroauration of a range of substituted alkynes to give (Z)-vinylgold complexes (C^N^C)Au–C(R^1^)=CH(R^2^) with almost quantitative stereo- and regioselectivity. These reactions are tolerant of a large variety of functional groups including hydroxide and carboxylic acid functions.^8^ There is a growing realisation of the role that single-electron transfer steps and gold(ii) intermediates may play in gold-mediated reactions.^9^

We report here on the reactivity of *in situ* generated gold(ii) radicals with 1-alkenes, which leads to the formation of gold(iii) alkyl complexes. Alkyl complexes of C^N^C gold pincer complexes are accessible in a variety of ways: by alkylation with Grignard reagents or aluminium alkyls,^10–12^ by O-abstraction from (C^N^C)AuOMe with phosphines,^13^ or by the reaction of (C^N^C)AuOH with allylic alcohols,^13^ or by the reaction of (C^N^C)AuCl/base with C–H acidic alkanes CH_2_R^1^R^2^. This last method is very versatile and gives alkyls (C^N^C)AuCHR^1^R^2^ which carry functional groups in the α-position.^12^ Here we describe the hydroauration of alkenes to give gold(iii) alkyls with functional groups in β-position. The experimental and computational results provide insights into the factors influencing radical-based hydroaurations of unsaturated substrates.

## Results and discussion

For solubility reasons, from the library of differently substituted C^N^C gold(iii) hydrides previously reported,^1,8^ we chose to carry out the reactions reported here using the *p*-OMe substituted gold hydride, (C^N^OMe^^C)AuH (1). This compound is accessible following literature procedures from (C^N^OMe^^C)AuCl and LiAlH_4_ in 85% yield.

**Scheme 2:**
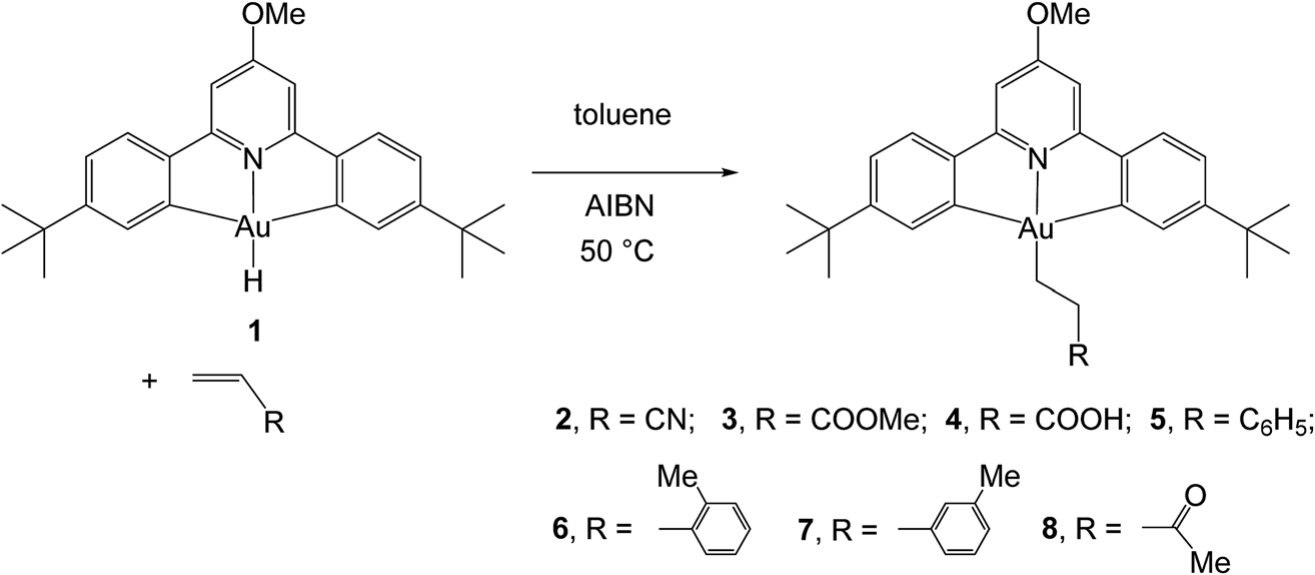
Synthesis of gold(iii) alkyls 2–8 from the gold(iii) hydride 1.

The reactivity of this complex towards different alkenes was investigated initially through scoping experiments carried out on a small scale, by mixing micromolar quantities of 1 and stoichiometric amounts of the alkene in toluene-*d*_8_ in a J-Young NMR tube (Scheme 2). Two molar equivalents of azobis*iso*butyronitrile (AIBN) were added, the mixture was shaken and heated in the dark to 50 °C to induce the decomposition of AIBN. The progress of the alkene hydroaurations was monitored by ^1^H NMR spectroscopy. At the end of the reaction the volatile components were removed *in vacuo*, the residue was washed with *n*-hexane followed by MeOH to remove any unreacted alkene and excess AIBN, and the residue was dissolved in CD_2_Cl_2_. The product was characterized spectroscopically. This method led to the formation of the alkyls 2–8 in high yields.

For the alkenes CH_2_=CHR [R = CN, COOMe, COOH, Ph, 2-MeC_6_H_4_, 3-MeC_6_H_4_, C(O)Me] this resulted in the clean and facile formation of the corresponding gold-alkyl products 2–8. These reactions were also conducted on a preparative scale and allowed the isolation of the gold alkyls 2–8 as microcrystalline powders in moderate yields, with losses being mainly due to the washing steps during purification. Attempts to obtain crystals suitable for X-ray diffraction were unfortunately not successful.

However, another series of alkenes gave slow reactions that led to mixtures of products which could not be purified. This behaviour was shown by unfunctionalised alkenes (1-pentene and 1-hexene), by allylic derivatives CH_2_=CHR (R = CH_2_OH, CH_2_NH_2_, CH_2_COOH), and by more highly substituted and internal alkenes, notably 1,1-diphenylethene, α-methylstyrene, *p-tert*-butylstyrene, 2-methyl-3-butenol and 3-pentenoic acid. Finally, *cis*- and *trans*-stilbene, *cis*- and *trans*-2-pentene and cyclopentene proved entirely unreactive.

**Scheme 3:**
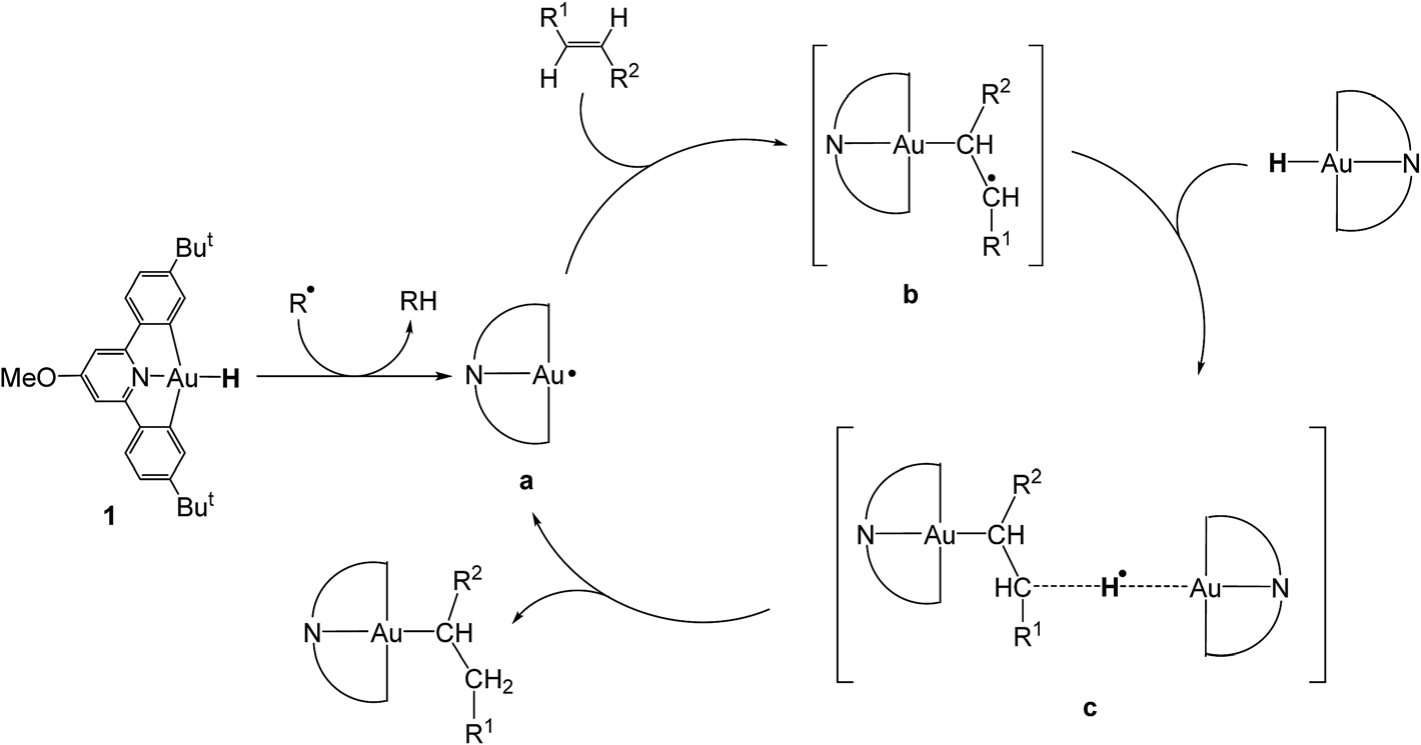
Proposed mechanism for the radical-initiated hydroauration of alkenes by 1.

There were therefore two classes of alkenes: those that gave clean insertions into the Au–H bond, and those that showed borderline or no reactivity. In order to rationalize the reactivity differences observed for the various alkene substrates, a computational investigation was undertaken using density functional theory (DFT).^14^ It is proposed that the mechanism of alkene hydroauration follows the principles previously established for the correspondent alkyne reactions,^8^ as shown in Scheme 3, and involves various radical intermediates a, b and c.

Our calculations focussed on the first step of the mechanism described above, the formation of the intermediate radical species b* from the gold(ii) radical a* and the alkene substrate. The model for the pincer ligand was simplified by omitting the Bu^*t*^ and OMe substituents (denoted by *). The energetics of this reaction step were investigated by calculating the standard reaction enthalpies 
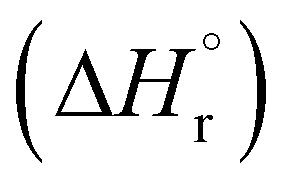
, Gibbs free energy of reaction 
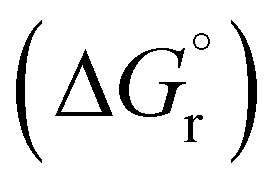
 and the total electronic energy differences (Δ*E*_tot_) in the gas phase and under standard conditions.

Very similar trends were calculated for the three parameters taken into consideration (see Table 1). In particular, the calculated values of 
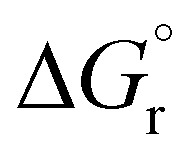
 reflect the experimental observations quite accurately: the formation of alkyl radicals b1*–b5* is energetically favourable, as observed by the clean, near-quantitative formation of gold alkyls in these cases, while reactions leading to b6*–b15* are close to 
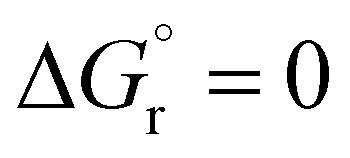
 or positive and are therefore not predicted to proceed.

**Table 1:** Standard reaction enthalpies 
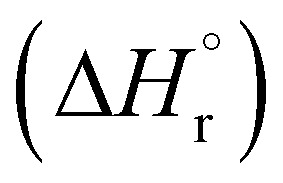
, Gibbs free energy of reaction 
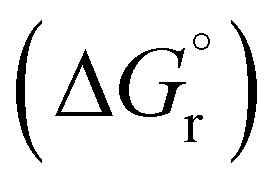
, and total electronic energy differences (Δ*E*_tot_) calculated in the gas phase at 298 K for the formation of gold(iii) alkyl, vinyl and allenyl radicals from the corresponding unsaturated substrates and a* (Scheme 3)

Radical	R	R′	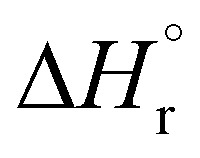 (kcal mol^−1^)	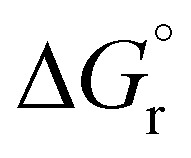 (kcal mol^−1^)	Δ*E*_tot_ (kcal mol^−1^)
b1*	CN	H	−18.68	−6.52	−19.61
b2*	COOMe	H	−16.84	−4.25	−17.86
b3*	COOH	H	−17.03	−4.47	−18.04
b4*	Ph	H	−18.50	−5.79	−19.34
b5*	C(O)Me	H	−17.85	−5.11	−19.04
b6*	CH_2_OH	H	−13.59	−1.31	−14.07
b7*	CH_2_NH_2_	H	−11.68	0.66	−12.34
b8*	CH_2_COOH	H	−13.39	−0.49	−14.00
b9*	*n*-Pr	H	−11.32	0.92	−11.83
b10*	*n*-Bu	H	−11.26	0.87	−11.81
b11*	Ph	Ph	−11.70	1.94	−12.30
b12*	Me	Et	−7.33	4.89	−7.95
b13*	Et	Me	−7.56	4.36	−8.06
b14*	Me	CH_2_COOH	−8.32	4.78	−8.84
b15*	CH_2_COOH	Me	−8.24	4.28	−9.12
d1*	Ph	H	−23.20	−12.48	−23.94
d2*	Ph	Ph	−17.47	−6.60	−17.98
d3*	*n*-Bu	H	−16.67	−5.17	−17.65
d4*	Ph	Me	−17.11	−6.09	−18.36
d5*	SiMe_3_	H	−17.46	−6.36	−18.48
h1*	Me	Me	−29.18	−16.22	−29.23

**Scheme 4:**
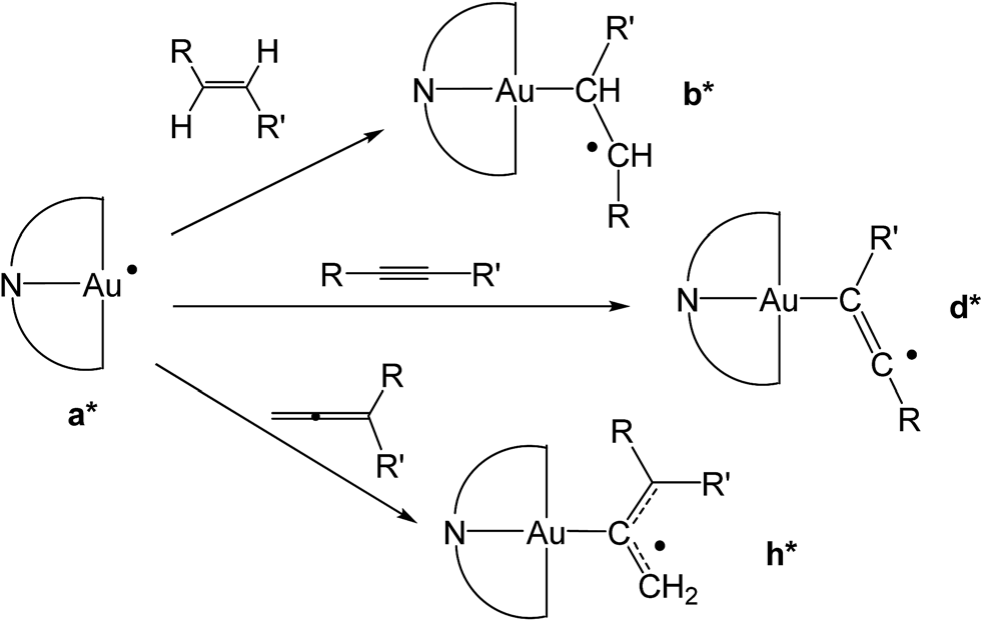
Formation of differently substituted alkyl, vinyl and allyl radicals through the reaction of the gold(ii) species a* with alkene, alkyne and allene substrates, respectively.

**Fig. 1:**
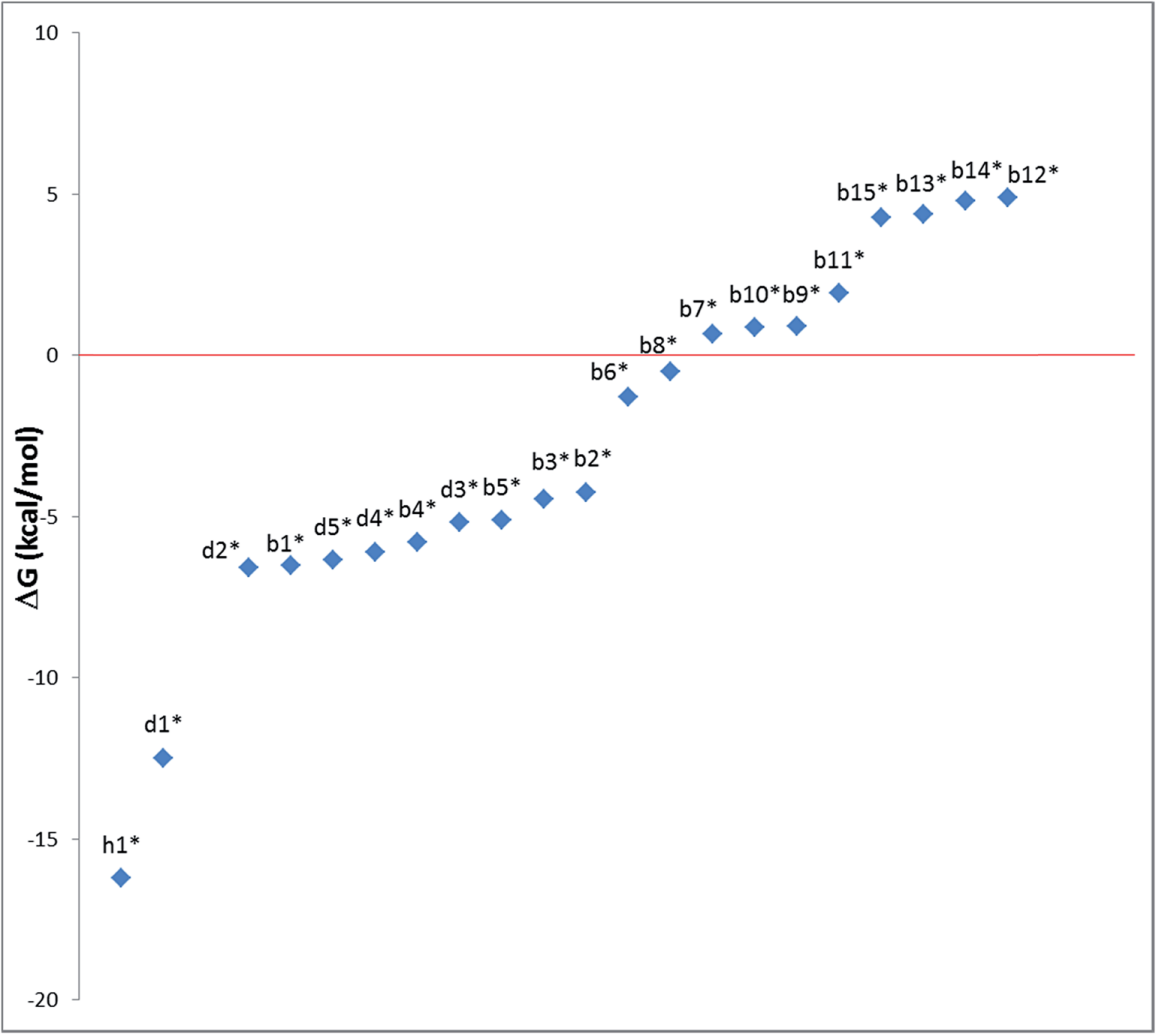
Trend of 
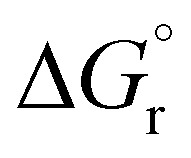
, (*T* = 298 K) calculated for the reactions depicted in Scheme 3 (kcal mol^−1^). The substituents for the radical species b1*–b15*, d1*–d5* and h1* are as listed in Table 1.

To provide a further insight into the observed trends, the calculations were extended to the previously investigated alkyne^8^ and allene^1^ substrates, and in particular to the formation of some of the corresponding vinyl and allyl radicals d* and h*, respectively, from the reaction with a* (Scheme 4). For all of these systems, the calculated values of 
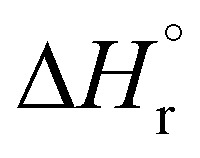
, Δ*E*_tot_ and 
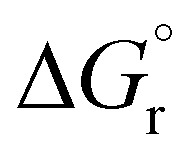
 were more negative than in the case of the alkene substrates (Table 1 and Fig. 1). The reaction with allenes to give the allyl radical h1* proved energetically particularly favourable, in agreement with the experimentally observed facile hydroauration of allenes by (C^N^C)AuH.^1^ The formation of the vinyl radical d1* is also strongly exergonic, while there is little energy difference between the other mono- and disubstituted alkynes in this series.

**Scheme 5:**
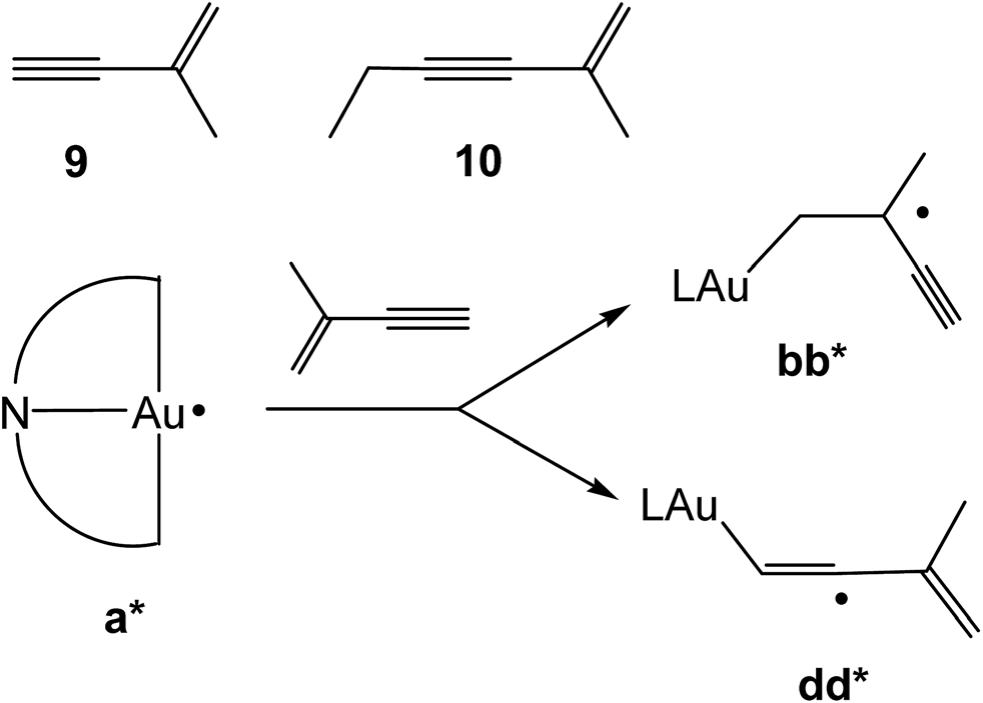
Reactions with enynes.

The chemoselectivity of the hydroauration was explored using the enynes 9 and 10 (Scheme 5), under analogous AIBN-initiated conditions. NMR spectroscopy showed that product mixtures are formed from attack on both the double and triple bonds, which in the case of 9 occurred with about equal probability, while 10 gave an approximately 80 : 20 mixture with predominant attack on C=C. In agreement with this, calculations of the reaction of species a* with 2-methylbuten-3-yne showed essentially identical Δ*E*_tot_ values for the formation of bb* (Δ*E*_tot_ = −26.06 kcal mol^−1^) and dd* (Δ*E*_tot_ = −26.11 kcal mol^−1^) (Scheme 5).

**Fig. 2:**
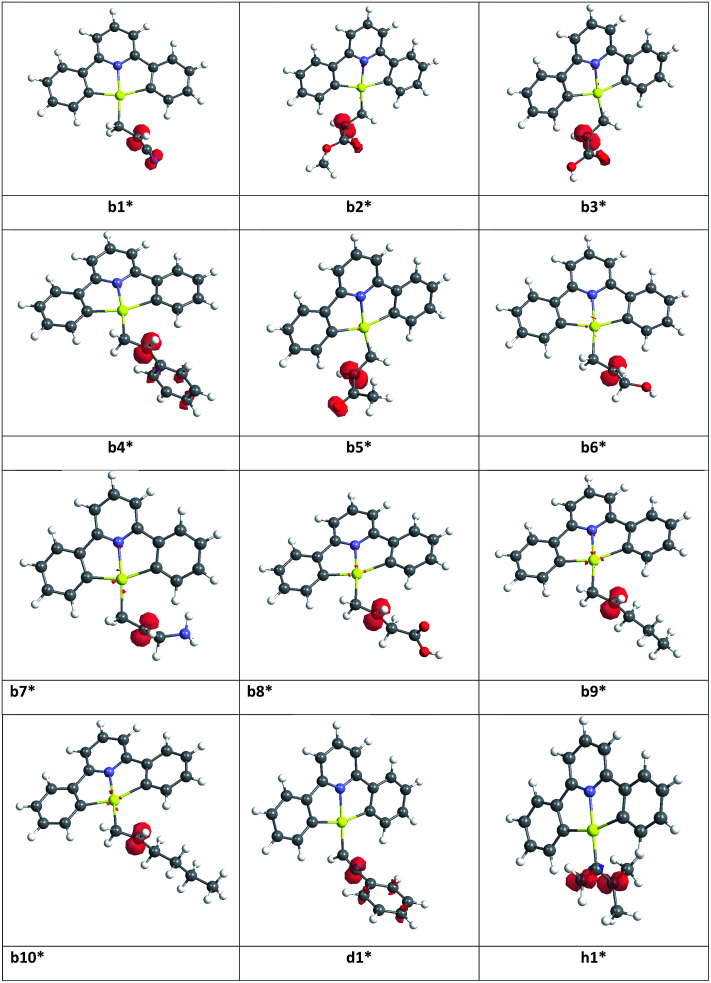
Spin density maps for radical species b1*–b10*, d1* and h1*.

In order to rationalize observed alkene reactivity pattern, an investigation of the spin density in different b*, d* and h* radical intermediates was performed. As summarized in Fig. 2, in radicals b1*–b5*, d1*, and h1* a significant degree of spin delocalization is observed, while such delocalization does not arise in b6*–b10*. This suggests that the reason for the energy difference in the formation of radicals on reaction with the gold(ii) species reflects these differences in spin delocalisation, the most stable radical intermediates being those stabilized by resonance. Accordingly, all of the alkyne and allene substrates previously explored,^1,8^ whose radicals can in all cases be stabilized by resonance, were observed to undergo facile hydroauration, while for the alkenes the reactivity depends on the nature of the substituent.

## Conclusion

The hydroauration of 1-alkenes with the gold(iii) hydride pincer complex (C^N^OMe^^C)AuH is initiated by radicals and appears to follow the same bimolecular outer-sphere mechanism that has previously been established for the regio- and stereoselective hydroauration of alkynes. The process involves the generation of a (C^N^C)Au(ii) radical which reacts with alkenes to give a gold-substituted alkyl radical. According to DFT calculations, the determining factor for the reaction appears to be the energy change associated with the attack by a gold(ii) radical species on the alkene. Alkenes leading to alkyl radicals with restricted spin delocalisation either reacted slowly to a mixture of products, or did not react at all. While this limits the scope of the method to some extent, the hydroauration of activated alkenes is a facile method for the metal alkyl-free generation of gold(iii) alkyl complexes bearing a variety of functional groups in β-position, including cyano, keto, ester and carboxylic acid functions. Moreover, the present study suggests that the hydroauration by gold(iii) hydrides can be extended to different classes of unsaturated species, and that the reactivity trend of different substrates can be rationalized and/or predicted based on the spin delocalization of the radical intermediates involved. The scope of such alkyls for C(sp^2^)–C(sp^3^) coupling reactions by reductive elimination using (aryl)(alkyl)gold(iii) complexes is currently under investigation.

## Experimental

When required, manipulations were performed by using standard Schlenk techniques under dry nitrogen or a MBraun glove box. Nitrogen was purified by passing through columns of supported P_2_O_5_ with moisture indicator, and of activated 4 Å molecular sieves. Anhydrous solvents were freshly distilled from appropriate drying agents. Elemental analyses were carried out at London Metropolitan University. AIBN (BDH Chemicals) was degassed by evacuation and stored under N_2_ in the glovebox before use. The alkenes (Sigma Aldrich) were degassed by freeze–pump–thaw cycles and stored over activated 4 Å molecular sieves before use. Solvents, toluene-*d*_8_ and CD_2_Cl_2_ (Apollo Scientific) were degassed by three freeze–pump–thaw cycles and stored over activated 4 Å molecular sieves prior to use. ^1^H and ^13^C{^1^H} NMR spectra were recorded using a Bruker Avance DPX-300 spectrometer equipped with a ^1^H, BB smartprobe. ^1^H NMR spectra (300.13 MHz) were referenced to the residual protons of the deuterated solvent used. ^13^C{^1^H} NMR spectra (75.47 MHz) were referenced to the D-coupled ^13^C resonances of the NMR solvent.

### Preparation of (C^N^OMe^^C)AuH (1)

Complex 1 was prepared by a modification of a literature procedure.^8^ Under a N_2_ atmosphere, 0.40 g (0.66 mmol) of the chloro complex (C^N^OMe^^C)AuCl was charged in a dry Schlenk flask with 40 mL of dry toluene. The mixture was cooled to −78 °C and a solution of LiAlH_4_ in dry THF (0.05 M, 13 mL, 0.66 mmol) was added dropwise. The mixture was stirred at −78 °C in the dark for 15 min, yielding a dark suspension which was filtered under N_2_. The filtrate was evaporated to dryness to afford a brown powder. This was taken up in dichloromethane, and the resulting suspension was filtered over cotton in the dark, to give a pale-yellow filtrate. The solvent was removed under reduced pressure, and a white solid was obtained, yield 0.38 g (0.56 mmol, 85%).

### Synthesis and characterization of insertion products 2–8

#### NMR scale reactions

As a general procedure, a solution of 1 (0.009 mmol) in toluene-*d*_8_ (0.7 mL) was prepared inside a glovebox in a J-Young NMR tube. The desired olefin (1 molar equivalent) was then added using a microlitre syringe, followed by 2 equivalents of AIBN. The tube was shaken and heated in the dark to 50 °C. The reactions were monitored by ^1^H NMR spectroscopy. The volatile components were removed *in vacuo* at the end of the reaction, and the residue was washed with *n*-hexane and with MeOH, and eventually redissolved in CD_2_Cl_2_ for the NMR characterization. Yields were calculated from the NMR integration.

#### (C^N^OMe^^C)Au(CH_2_)_2_CN (2)

This compound was synthesized from 1 (5.0 mg, 0.009 mmol), acrylonitrile (6 μL of a 1.5 M solution in toluene-*d*_8_, 0.009 mmol) and 3 mg of AIBN (0.018 mmol). The reaction was complete after 75 min at 50 °C. Yield: 80%.

#### (C^N^OMe^^C)Au(CH_2_)_2_COOMe (3)

This compound was made from 1 (5.0 mg, 0.009 mmol), methyl acrylate (6 μL of a 1.5 M solution in toluene-*d*_8_, 0.009 mmol) and 3 mg of AIBN (0.018 mmol). Conversion was complete after 50 min at 50 °C. Yield: 90%.

#### (C^N^OMe^^C)Au(CH_2_)_2_COOH (4)

This compound was made from 1 (5.0 mg, 0.009 mmol), acrylic acid (10 μL of a 0.9 M solution in toluene-*d*_8_, 0.009 mmol) and 3 mg of AIBN (0.018 mmol). Conversion was complete after 30 min at 50 °C. Yield: 90%.

#### (C^N^OMe^^C)Au(CH_2_)_2_Ph (5)

This compound was made from 1 (5.0 mg, 0.009 mmol), styrene (10 μL of a 0.9 M solution in toluene-*d*_8_, 0.009 mmol) and 3 mg of AIBN (0.018 mmol). Conversion was complete after 10 h at 50 °C. Yield: 85%.

#### (C^N^OMe^^C)Au(CH_2_)_2_(2-MeC_6_H_4_) (6)

This compound was made from 1 (5.0 mg, 0.009 mmol), 2-methylstyrene (10 μL of a 0.9 M solution in toluene-*d*_8_, 0.009 mmol) and 3 mg of AIBN (0.018 mmol). Conversion was complete after 31 h at 50 °C. Yield: 90%.

#### (C^N^OMe^^C)Au(CH_2_)_2_(3-MeC_6_H_4_) (7)

This compound was made from 1 (5.0 mg, 0.009 mmol), 3-methylstyrene (10 μL of a 0.9 M solution in toluene-*d*_8_, 0.009 mmol) and 3 mg of AIBN (0.018 mmol). Conversion was complete after 13 h at 50 °C. Yield: 90%.

#### (C^N^OMe^^C)Au(CH_2_)_2_C(O)Me (8)

This compound was made from 1 (5.0 mg, 0.009 mmol), 3-buten-2-one (10 μL of a 0.9 M solution in toluene-*d*_8_, 0.009 mmol) and 3 mg of AIBN (0.018 mmol). Conversion was complete after 5 h at 50 °C. Yield: 90%.

#### Reactions on a preparative scale

As a general procedure, to a solution of 1 (0.07 mmol) in dry and degassed toluene (4 mL) under nitrogen was added 1 molar equivalent of the desired olefin using a microlitre syringe, followed by 1 equivalent of AIBN. The mixture was heated in the dark to 50 °C. The reactions were monitored by ^1^H NMR spectroscopy. The volatile components were removed *in vacuo* at the end of the reaction, and the residue was washed with *n*-hexane and with MeOH.

#### (L^OMe^)Au(CH_2_)_2_CN (2)


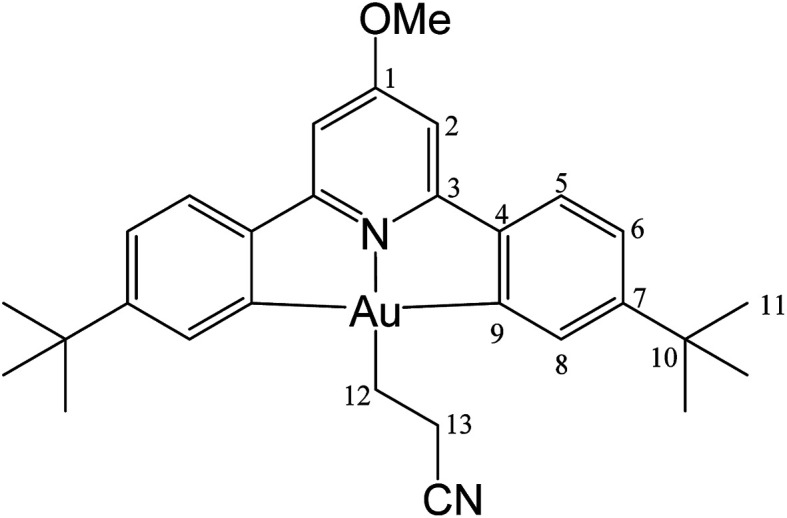
The compound was synthesized from 1 (40.0 mg, 0.07 mmol), acrylonitrile (5 μL, 0.07 mmol) and 11.0 mg of AIBN (0.007 mmol), and the reaction resulted complete after 2 h at 50 °C. Yield: 20.0 mg (0.03 mmol), 45%. Anal. calcd for C_29_H_33_AuN_2_O: C, 55.95; H, 5.34; N, 4.50. Found: C, 55.41; H, 5.11; N, 5.04. ^1^H-NMR (300.13 MHz, CD_2_Cl_2_): 7.70 (d, 2H, ^4^*J* = 1.8 Hz, H8), 7.57 (d, 2H, ^3^*J* = 8.1 Hz, H5), 7.30 (dd, 2H, ^3^*J* = 8.1, ^4^*J* = 1.8 Hz, H6), 6.96 (s, 2H, H2), 4.01 (s, 3H, OMe), 2.89 (t, 2H, ^3^*J* = 8.0 Hz, H12), 2.08 (t, 2H, ^3^*J* = 8.0 Hz, H13), 1.38 ppm (s, 18H, H11). ^13^C{^1^H} NMR (75.47 MHz, CD_2_Cl_2_): *δ* 170.6 (s, C1), 166.7 (s, C9), 164.4 (s, C3), 154.4 (s, C7), 148.2 (s, C4), 130.1 (s, C8), 125.2 (s, C5), 123.7 (s, C6), 122.2 (s, CN), 102.4 (s, C2), 56.5 (s, OMe), 35.6 (s, C10), 31.4 (s, C11), 18.9 (s, C12), 16.7 ppm (s, C13).

#### (C^N^OMe^^C)Au(CH_2_)_2_COOMe (3)


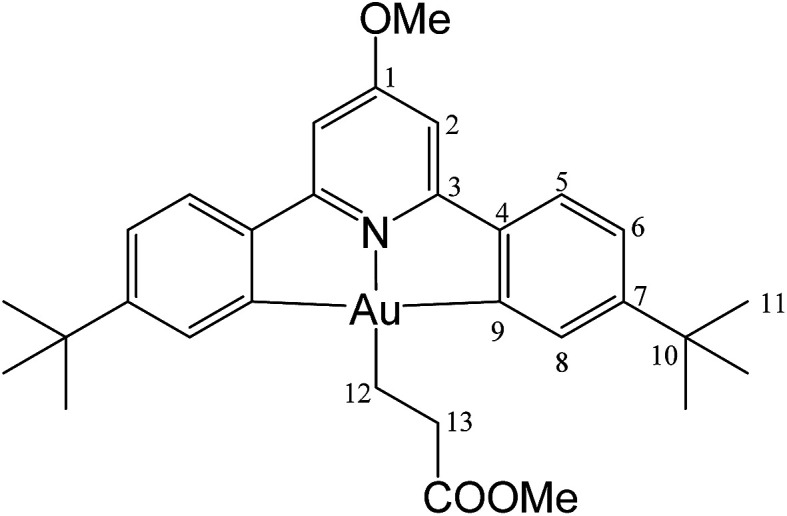
This compound was synthesized from 1 (40.0 mg, 0.07 mmol), methyl acrylate (4 μL, 0.07 mmol) and 11.0 mg of AIBN (0.007 mmol). The reaction was complete after 70 min at 50 °C. Yield: 20.0 mg (0.03 mmol), 44%. Anal. calcd for C_30_H_36_AuNO_3_: C, 54.96; H, 5.54; N, 2.14. Found: C, 54.23; H, 5.78; N, 2.68. ^1^H-NMR (300.13 MHz, CD_2_Cl_2_): *δ* 7.75 (d, 2H, ^4^*J* = 1.6 Hz, H8), 7.55 (d, 2H, ^3^*J* = 8.1 Hz, H5), 7.27 (dd, 2H, ^3^*J* = 8.1, ^4^*J* = 1.6 Hz, H6), 6.95 (s, 2H, H2), 4.00 (s, 3H, OMe), 3.59 (s, 3H, COOMe), 2.84 (t, 2H, ^3^*J* = 8.0 Hz, H12), 2.10 (t, 2H, ^3^*J* = 8.0 Hz, H13), 1.38 ppm (s, 18H, H11). ^13^C{^1^H} NMR (75.47 MHz, CD_2_Cl_2_): *δ* 175.7 (s, *C*OOMe), 170.6 (s, C1), 167.3 (s, C9), 164.3 (s, C3), 154.2 (s, C7), 148.2 (s, C4), 130.4 (s, C8), 125.0 (s, C5), 123.4 (s, C6), 102.4 (s, C2), 56.4 (s, OMe), 51.6 (s, COO*Me*), 35.2 (s, C10), 31.4 (s, C11), 25.4 (s, C12), 18.1 ppm (s, C13).

#### (C^N^OMe^^C)Au(CH_2_)_2_COOH (4)


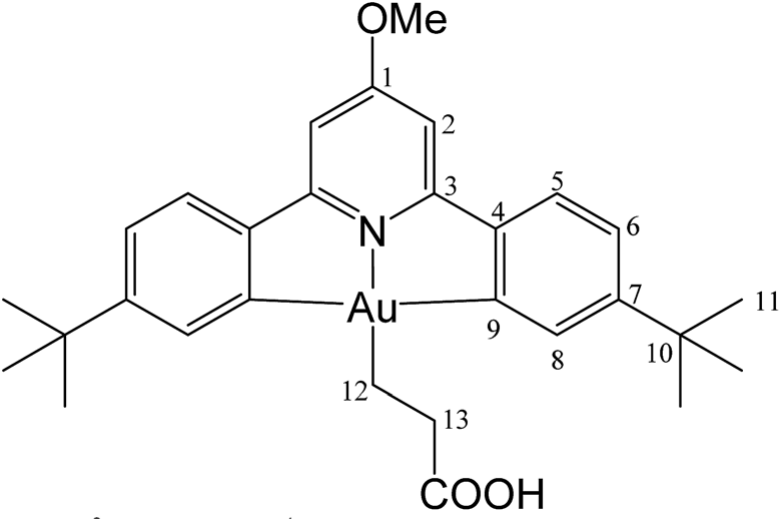
This compound was synthesized from 1 (40.0 mg, 0.07 mmol), acrylic acid (4 μL, 0.07 mmol) and 11.0 mg of AIBN (0.007 mmol), and the reaction resulted complete after 80 min at 50 °C. Yield: 20.5 mg (0.04 mmol), 56%. Anal. calcd for C_29_H_34_AuNO_3_: C, 54.29; H, 5.34; N, 2.18. Found: C, 53.73; H, 5.45; N, 2.76. ^1^H-NMR (300.13 MHz, CD_2_Cl_2_): *δ* 7.78 (d, 2H, ^4^*J* = 1.4 Hz, H8), 7.56 (d, 2H, ^3^*J* = 8.2 Hz, H5), 7.28 (dd, 2H, ^3^*J* = 8.2, ^4^*J* = 1.4 Hz, H6), 6.97 (s, 2H, H2), 4.01 (s, 3H, OMe), 2.89 (t, 2H, ^3^*J* = 8.0 Hz, H12), 2.13 (t, 2H, ^3^*J* = 8.0 Hz, H13), 1.38 ppm (s, 18H, H11). ^13^C{^1^H} NMR (75.47 MHz, CD_2_Cl_2_): *δ* 179.3 (s, COOH), 170.7 (s, C1), 167.2 (s, C9), 164.4 (s, C3), 154.4 (s, C7), 148.3 (s, C4), 130.4 (s, C8), 125.1 (s, C5), 123.5 (s, C6), 102.4 (s, C2), 56.5 (s, OMe), 35.6 (s, C10), 31.4 (s, C11), 25.4 (s, C12), 17.4 ppm (s, C13).

#### (L^OMe^)Au(CH_2_)_2_Ph (5)


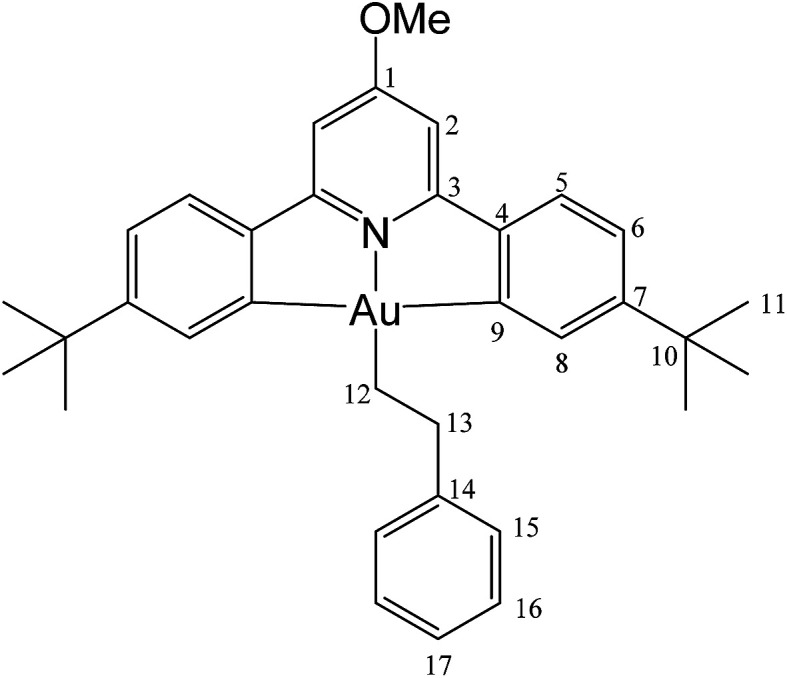
This compound was synthesized from 1 (40.0 mg, 0.07 mmol), styrene (4 μL, 0.07 mmol) and 11.0 mg of AIBN (0.007 mmol). The reaction was complete after 8 h at 50 °C. Yield: 6.0 g (0.009 mmol), 13%. Anal. calcd for C_34_H_38_AuNO: C, 60.62; H, 5.69; N, 2.08. Found: C, 59.92; H, 5.78; N, 2.48. ^1^H-NMR (300.13 MHz, CD_2_Cl_2_): *δ* 7.81 (d, 2H, ^4^*J* = 1.8 Hz, H8), 7.54 (d, 2H, ^3^*J* = 8.1 Hz, H5), 7.38 (d, 2H, ^3^*J* = 7.4 Hz, H15), 7.29 (m, 4H, H6 + H16), 7.16 (t, 1H, ^3^*J* = 7.4 Hz, H17), 6.94 (s, 2H, H2), 3.99 (s, 3H, OMe), 3.16 (t, 2H, ^3^*J* = 8.2 Hz, H12), 2.14 (t, 2H, ^3^*J* = 8.2 Hz, H13), 1.40 ppm (s, 18H, H11). ^13^C{^1^H} NMR (75.47 MHz, CD_2_Cl_2_): *δ* 170.6 (s, C1), 167.6 (s, C9), 164.2 (s, C3), 154.1 (s, C7), 148.4 (s, C4), 146.6 (s, C14), 130.4 (s, C8), 128.7 (s, C15 or C16), 128.7 (s, C15 or C16), 125.7 (s, C17), 125.0 (s, C5), 123.4 (s, C6), 102.4 (s, C2), 56.5 (s, OMe), 38.0 (s, C12), 35.6 (s, C10), 31.5 (s, C11), 27.6 ppm (s, C13).

#### (C^N^OMe^^C)Au(CH_2_)_2_(2-MeC_6_H_4_) (6)


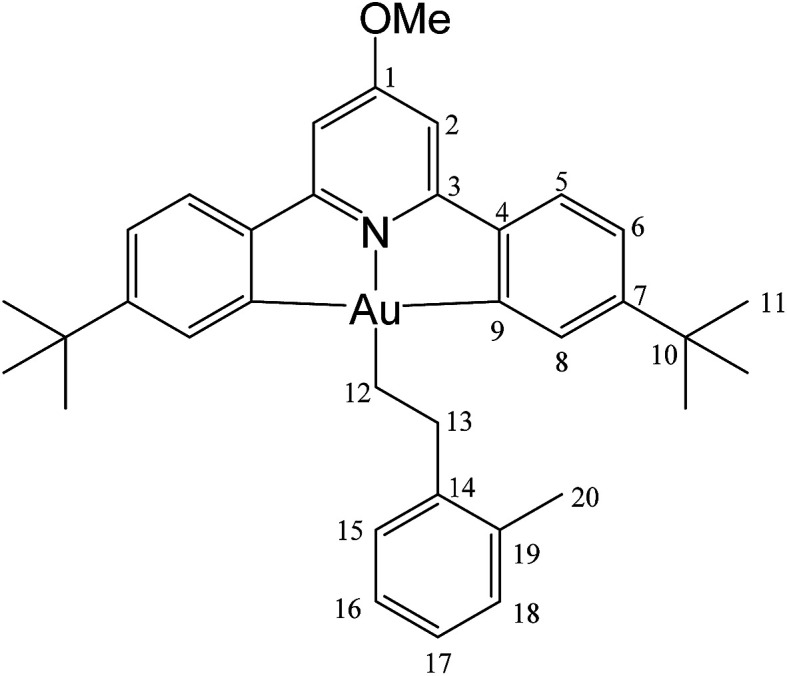
This compound was synthesized from 1 (40.0 mg, 0.07 mmol), 2-methylstyrene (6 μL, 0.07 mmol) and 11.0 mg of AIBN (0.007 mmol), and the reaction resulted complete after 6 h at 50 °C. Anal. yield: 19.0 g (0.027 mmol), 39%. Calcd for C_33_H_40_AuNO: C, 61.13; H, 5.86; N, 2.04. Found: C, 60.65; H, 5.36; N, 2.38. ^1^H-NMR (300.13 MHz, CD_2_Cl_2_): *δ* 7.85 (d, 2H, ^4^*J* = 1.8 Hz, H8), 7.57 (d, 2H, ^3^*J* = 8.2 Hz, H5), 7.35 (d, 2H, ^3^*J* = 7.2 Hz, H15), 7.28 (dd, 2H, ^3^*J* = 8.2, ^4^*J* = 1.8 Hz, H6), 7.14 (m, 2H, H16 + H18), 7.08 (t, 1H, ^3^*J* = 7.2 Hz, H17), 6.96 (s, 2H, H2), 4.0 (s, 3H, OMe), 3.15 (t, 2H, ^3^*J* = 8.7 Hz, H12), 2.51 (s, 3H, Me), 2.07 (t, 2H, ^3^*J* = 8.7 Hz, H13), 1.39 ppm (s, 18H, H11). ^13^C{^1^H} NMR (75.47 MHz, CD_2_Cl_2_): *δ* 170.6 (s, C1), 167.6 (s, C9), 164.2 (s, C3), 154.1 (s, C7), 148.4 (s, C4), 144.5 (s, C14), 135.9 (s, C19), 130.5 (s, C15), 130.5 (s, C8), 129.5 (C16), 125.6 (s, C17 or C18), 125.9 (C17 or C18), 125.1 (s, C5), 123.4 (s, C6), 102.4 (s, C2), 56.4 (s, OMe), 35.6 (s, C10), 35.2 (s, C12), 31.6 (s, C11), 25.9 (s, C13), 19.8 ppm (s, Me).

#### (C^N^OMe^^C)Au(CH_2_)_2_(3-MeC_6_H_4_) (7)


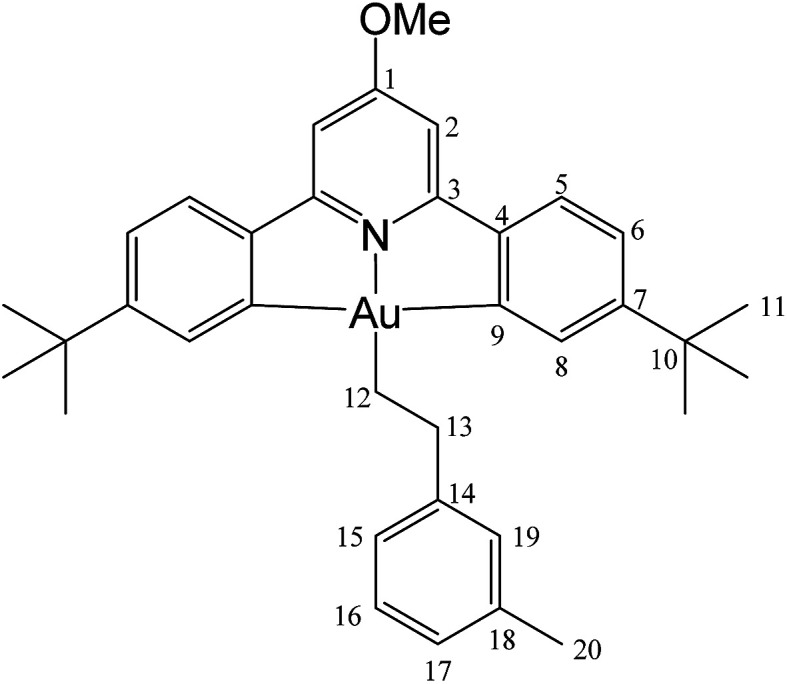
This compound was synthesized from 1 (40.0 mg, 0.07 mmol), 3-methylstyrene (9 μL, 0.07 mmol) and 11.0 mg of AIBN (0.007 mmol), and the reaction resulted complete after 8 h at 50 °C. Yield: 12.0 mg (0.02 mmol), 25%. Calcd for C_33_H_40_AuNO: C, 61.13; H, 5.86; N, 2.04. Found: C, 61.82; H, 5.97; N, 1.85. ^1^H-NMR (300.13 MHz, CD_2_Cl_2_): 7.83 (d, 2H, ^4^*J* = 1.9 Hz, H8), 7.58 (d, 2H, ^3^*J* = 8.1 Hz, H5), 7.29 (dd, 2H, ^3^*J* = 8.1, ^4^*J* = 1.9 Hz, H6), 7.20 (m, 3H, H15 + H17 + H19), 6.98 (m, 3H, H2 + H16), 4.0 (s, 3H, OMe), 3.11 (t, 2H, ^3^*J* = 8.1 Hz, H12), 2.33 (s, 3H, Me), 2.14 (t, 2H, ^3^*J* = 8.1 Hz, H13), 1.40 ppm (s, 18H, H11). ^13^C{^1^H} NMR (75.47 MHz, CD_2_Cl_2_): *δ* 170.6 (s, C1), 167.6 (s, C9), 164.2 (s, C3), 154.1 (s, C7), 148.4 (s, C4), 146.5 (s, C14), 138.2 (s, C19), 130.4 (s, C8), 129.6 (s, C15), 128.6 (C16), 126.5 (s, C17 or C18), 125.6 (C17 or C18), 125.1 (s, C5), 123.4 (s, C6), 102.4 (s, C2), 56.4 (s, OMe), 37.9 (s, C12), 35.6 (s, C10), 31.5 (s, C11), 27.7 (s, C13), 21.6 ppm (s, Me).

#### (C^N^OMe^^C)Au(CH_2_)_2_C(O)Me (8)


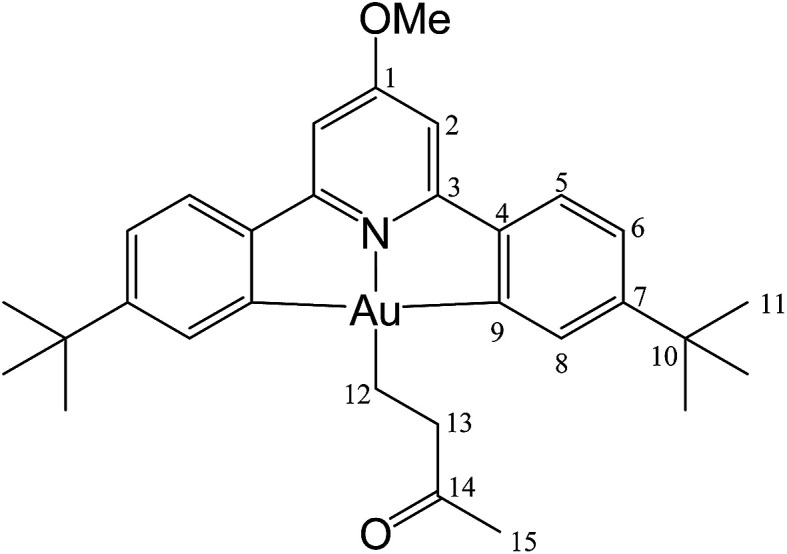
This compound was synthesized from 1 (40.0 mg, 0.07 mmol), 3-buten-2-one (6 μL, 0.07 mmol) and 11.0 mg of AIBN (0.007 mmol). The reaction was complete after 60 min at 50 °C. Yield: 35.0 mg (0.06 mmol), 78%. Anal. calcd for C_30_H_36_AuNO_2_: C, 56.34; H, 5.67; N, 2.19. Found: C, 56.21; H, 5.57; N, 2.30. ^1^H-NMR (300.13 MHz, CD_2_Cl_2_): *δ* 7.74 (d, 2H, ^4^*J* = 1.4 Hz, H8), 7.57 (d, 2H, ^3^*J* = 8.2 Hz, H5), 7.28 (dd, 2H, ^3^*J* = 8.2, ^4^*J* = 1.4 Hz, H6), 6.97 (s, 2H, H2), 4.01 (s, 3H, OMe), 2.93 (t, 2H, ^3^*J* = 8.1 Hz, H12), 2.93 (s, 3H, Me), 2.20 (t, 2H, ^3^*J* = 8.1 Hz, H13), 1.37 ppm (s, 18H, H11). ^13^C{^1^H} NMR (75.47 MHz, CD_2_Cl_2_): *δ* 210.6 (s, *C*(O)Me), 170.6 (s, C1), 167.3 (s, C9), 164.2 (s, C3), 154.2 (s, C7), 148.3 (s, C4), 130.3 (s, C8), 125.1 (s, C5), 123.5 (s, C6), 102.4 (s, C2), 56.4 (s, OMe), 45.4 (s, C(O)*Me*), 35.6 (s, C10), 31.4 (s, C11), 29.6 (s, C12), 17.4 ppm (s, C13).

### DFT calculations

Theoretical calculations were performed at the Density Functional Theory (DFT) level^14^ using the Gaussian 09 package of programs.^15^ Geometry optimizations were carried out using the PBE0 (PBE1PBE) hybrid functional,^16^ along with the Schäfer, Horn, and Ahlrichs double-ζ plus polarization all-electron basis sets^17^ for all atomic species. The nature of the minima of each optimized structure was verified by harmonic frequency calculations. The software Chemissian^18^ was used for the preparation of spin density distribution figures.

## Conflicts of interest

The authors declare no conflict of interest.

## Supplementary Material

RA-008-C7RA13481A-s001

## References

[cit1] Roşca D.-A., Smith D. A., Hughes D. L., Bochmann M. (2012). Angew. Chem., Int. Ed..

[cit2] Roşca D.-A., Wright J. A., Bochmann M. (2015). Dalton Trans..

[cit3] Kumar R., Nevado C. (2017). Angew. Chem., Int. Ed..

[cit4] Savjani N., Roşca D.-A., Schormann M., Bochmann M. (2013). Angew. Chem., Int. Ed..

[cit5] Rocchigiani L., Fernandez-Cestau J., Agonigi G., Chambrier I., Budzelaar P. H. M., Bochmann M. (2017). Angew. Chem., Int. Ed..

[cit6] Roşca D.-A., Fernandez-Cestau J., Morris J., Wright J. A., Bochmann M. (2015). Sci. Adv..

[cit7] Roşca D. –A., Wright J. A., Hughes D. L., Bochmann M. (2013). Nat. Commun..

[cit8] Pintus A., Rocchigiani L., Fernandez-Cestau J., Budzelaar P. H. M., Bochmann M. (2016). Angew. Chem., Int. Ed..

[cit9] Sahoo B., Hopkinson M. N., Glorius F. (2013). J. Am. Chem. Soc..

[cit10] Smith D. A., Roşca D.-A., Bochmann M. (2012). Organometallics.

[cit11] To W.-P., Tong G. S. M., Cheung C.-W., Yang C., Zhoua D., Che C.-M. (2017). Inorg. Chem..

[cit12] Fernandez-Cestau J., Bertrand B., Pintus A., Bochmann M. (2017). Organometallics.

[cit13] Chambrier I., Roşca D.-A., Fernandez-Cestau J., Hughes D. L., Budzelaar P. H. M., Bochmann M. (2017). Organometallics.

[cit14] Koch W. and Holthausen M. C., A Chemist's Guide to Density Functional Theory, Wiley-VCH, Weinheim, Germany, 2nd edn, 2002

[cit15] Frisch M. J. , Trucks G. W., Schlegel H. B., Scuseria G. E., Robb M. A., Cheeseman J. R., Scalmani G., Barone V., Mennucci B., Petersson G. A., Nakatsuji H., Caricato M., Li X., Hratchian H. P., Izmaylov A. F., Bloino J., Zheng G., Sonnenberg J. L., Hada M., Ehara M., Toyota K., Fukuda R., Hasegawa J., Ishida M., Nakajima T., Honda Y., Kitao O., Nakai H., Vreven T., Montgomery Jr J. A., Peralta J. E., Ogliaro F., Bearpark M., Heyd J. J., Brothers E., Kudin K. N., Staroverov V. N., Keith T., Kobayashi R., Normand J., Raghavachari K., Rendell A., Burant J. C., Iyengar S. S., Tomasi J., Cossi M., Rega N., Millam J. M., Klene M., Knox J. E., Cross J. B., Bakken V., Adamo C., Jaramillo J., Gomperts R., Stratmann R. E., Yazyev O., Austin A. J., Cammi R., Pomelli C., Ochterski J. W., Martin R. L., Morokuma K., Zakrzewski V. G., Voth G. A., Salvador P., Dannenberg J. J., Dapprich S., Daniels A. D., Farkas O., Foresman J. B., Ortiz J. V., Cioslowski J., Fox D. J., Gaussian 09, Revision C.01, Gaussian, Inc., Wallingford CT, 2010

[cit16] Adamo C., Barone V. (1999). J. Chem. Phys..

[cit17] Schäfer A., Horn H., Ahlrichs R. (1992). J. Chem. Phys..

[cit18] Skripnikov L. V. , Chemissian V. 4.43, Visualization Computer Program, http://www.chemissian.com, 2016

